# Genetic diversity of *Plasmodium falciparum* isolates from Baka Pygmies and their Bantu neighbours in the north of Gabon

**DOI:** 10.1186/s12936-015-0862-5

**Published:** 2015-10-09

**Authors:** Bertrand Mvé-Ondo, Dieudonné Nkoghe, Céline Arnathau, Virginie Rougeron, Ulrich Bisvigou, Lauriane Yacka Mouele, Larson Boundenga, Patrick Durand, Eric Elguero, Simone Lemmers, Lucrèce M. Délicat-Loembet, Nancy Diamella-Moukodoum, Christophe Paupy, François Renaud, Franck Prugnolle, Benjamin Ollomo

**Affiliations:** Centre International de Recherches Médicales de Franceville (CIRMF), BP 769, Franceville, Gabon; Ecole Doctorale Régionale d’Afrique Centrale en Infectiologie Tropicale, BP 876, Franceville, Gabon; Fac de Médecine, UMR-BIOMED, BP 8507, Libreville, Gabon; Ministry of Health, BP 50, Libreville, Gabon; Laboratoire Maladies Infectieuses et Vecteurs, Ecologie, Génétique, Evolution et Contrôle (MIVEGEC), UMR CNRS 5290/IRD 224, Université Montpellier, CHRU de Montpellier, 39 Avenue Charles Flahault, 34295 Montpellier, France; Département de Biologie Animale, Faculté des Sciences et Techniques, Laboratoire d’Écologie et Biologie évolutive, Université Cheikh AntaDiop de Dakar, BP 5005, Dakar, Senegal; Department of Anthropology, Durham University, Dawson Building, South Road, Durham, DH1 3LE England, UK

**Keywords:** Malaria, Genetic diversity, Population structure, Microsatellite loci, Antigenic determinants, Baka Pygmies, Bantus

## Abstract

**Background:**

There have been many reports on the population genetic structure of *Plasmodium falciparum* from different endemic regions especially sub-Saharan Africa. However, few studies have been performed on neglected populations, such as the Pygmy populations. In this study, the population genetic structure of *P. falciparum* was investigated in the Baka Pygmies of Gabon and compared to that observed in neighboring villages composed mostly of Bantu farmers.

**Methods:**

A total of 342 blood samples were collected from 170 Baka Pygmies and 172 Bantus in the north of Gabon (Woleu Ntem Province). *Plasmodium* infections were characterized by sequencing a portion of the parasite *cytochrome b* gene. Population genetic structure of *P. falciparum* in the different villages was analysed using microsatellite markers and genes coding for antigenic proteins (MSP1, MSP2, GLURP, and EBA-175).

**Results:**

Overall, prevalence of *P. falciparum* was around 57 % and no significant difference of prevalence was observed between Pygmies and Bantus. No significant differences of population genetic structure of *P. falciparum* was found between Pygmy and Bantu people except for one antigen-coding gene, *glurp*, for which genetic data suggested the existence of a potentially disruptive selection acting on this gene in the two types of populations. The genetic structure of *P. falciparum* followed a pattern of isolation by distance at the scale of the study.

**Conclusion:**

The prevalence and genetic diversity of *P. falciparum* observed in Baka demonstrates a significant transmission of the parasite in this population, and some exchanges of parasites with Bantu neighbours. Despite that, some antigen-coding genes seem to have had a particular evolutionary trajectory in certain Pygmy populations due to specific local human and/or mosquito characteristics.

**Electronic supplementary material:**

The online version of this article (doi:10.1186/s12936-015-0862-5) contains supplementary material, which is available to authorized users.

## Background

Malaria is the most important parasitic disease based on its impacts on human populations, with 207 million cases and 627,000 deaths recorded in 2012, especially in sub-Saharan Africa [1]. Currently, five species are known to infect humans (*Plasmodium falciparum, Plasmodium malariae*, *Plasmodium ovale*, *Plasmodium vivax,* and *Plasmodium knowlesi*). Among them, *P. falciparum* is the most virulent.

During the last 20 years, the genetic structure of *P. falciparum* populations has been intensely investigated all over the world because it provides information on the evolutionary potential of the parasite confronted by a new drug or a vaccine, on the risk of diffusion of resistance alleles throughout the world, as well as on its epidemiology and evolutionary history [2–11]. *Plasmodium falciparum* populations on the African continent, where most cases of infection occur every year, have largely been studied in this context [12–16]. However, some populations of this region have received very little attention regarding malaria infections. This is the case for instance for the Pygmy populations.

Pygmies are indigenous hunter-gatherers of the Central African forest who diverged from other groups in Africa about 60,000 years ago [17, 18]. They traditionally lived in small, nomad groups whose livelihood strategies were based on hunting, gathering, small-scale farming, and exchange of forest products with farming neighbours. Pygmies were traditionally intimately connected to the forest. Nowadays, Pygmy communities continue to maintain a strong link with the forest whenever possible, but the large majority have now abandoned their traditional way of life, spending more time in roadside settlements, with closer contacts with neighbouring Bantu farming communities, and more reliance on farming and wage labour [19, 20]. Because Pygmy populations remained isolated in forest ecosystems for a long time and are genetically divergent from the Bantu populations (thus constituting a different environment for the parasite), it is likely that their populations of parasites also remained isolated and developed specific genetic characteristics, especially at genes directly interacting with the host immune system.

In Gabon (Central Africa), there are three distinct Pygmy communities: the Baka, the Babongo and the Bakoya. None of them has conserved a traditional way of life even if some groups live in nomad forest camps for several months for hunting at certain periods of the year. In this country, most population genetic studies on malaria were conducted on the Bantu populations (urban and rural populations) [12, 21, 22]. Pygmies historically received limited attention from the health authorities and were largely neglected.

In this study, the population genetic structure of *P. falciparum* was characterized and compared among the Baka Pygmies and their Bantus neighbours in the same region of Gabon using both microsatellite and a set of antigen-coding genes.

## Methods

### Study area and studied populations

**Fig. 1:**
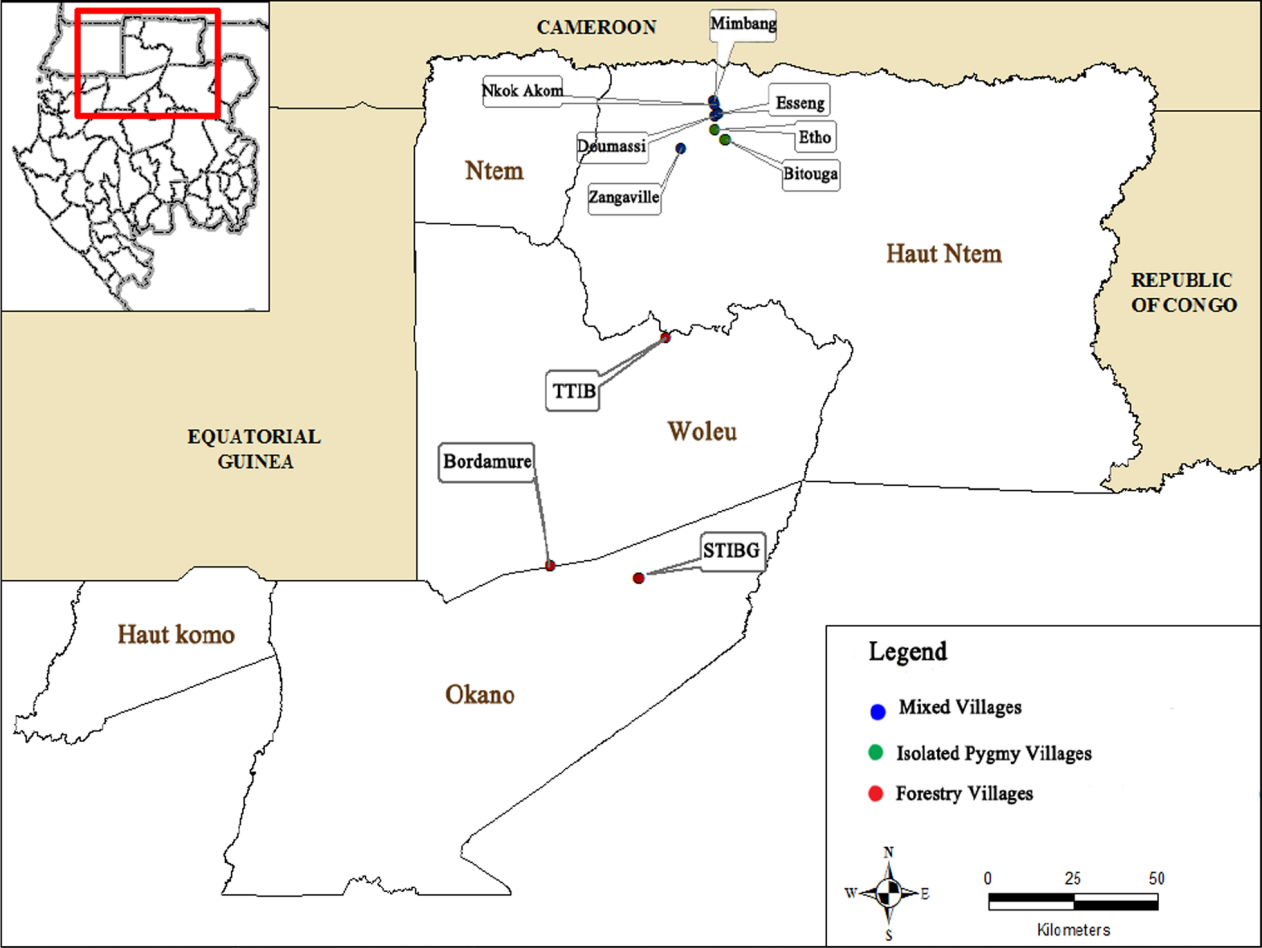
Geographical location of the study sites in the departments of Woleu and High Ntem in Woleu Ntem Province. Ten sites were sampled: (1) *green* isolated Pygmy villages; (2) *red* mixed villages (Bantus and Pygmies) and (3) *blue* Bantu populations in forestry villages

The study was carried out in Woleu Ntem Province, located in the north of Gabon (Fig. 1). Three countries surround this province: Cameroon, Equatorial Guinea and the Republic of Congo. The project focused on Baka Pygmies and Bantus living in rural and forest environments.

The study was conducted following two stages: (1) a sensitization stage in May 2013 during which persons were informed of the aim of the study, and, (2) blood sampling from June to July 2013. The sampling was conducted just after the rainy season (occurring from February to May) when malaria transmission is the highest.

Participants were recruited from ten villages (Fig. 1; Table 1): three in the Woleu region and seven in the region of Ntem Minvoul. Two villages (Bitouga and Etho) corresponded to what will be hereafter be referred as ‘isolated Pygmy villages’ (IPV). These villages were located in isolated forest zones, with no road access and were only composed by Pygmies. Five villages were ‘mixed villages’ (MV). Located along roads, these villages are mostly populated by Bantu peoples and a minority of Pygmy peoples. In these villages, both Bantus and Pygmies were sampled when possible (Table 1). Finally, three villages were villages built by forestry companies for their workers [hereafter named ‘forestry villages’ (FV)]. Only Bantu peoples lived in these villages, accessible by road. Distances between villages ranged from 1 to 136 km (Fig. 1). In mixed villages and other sites, participants were subjected to a questionnaire in which they would indicate their ethnic origin and the origin of their parents. Participants born from inter-ethnic marriages were not included in this study.

**Table 1:** Number of blood samples collected per village and ethnic group (Pygmies and Bantus) as well as prevalence (%) of *Plasmodium falciparum*

Collection sites and GPS positions	Pygmies		Bantus	
	Number of samples	Number of *P. falciparum* infections (prevalence %)	Number of samples	Number of *P. falciparum* infections (prevalence %)
Isolated Pygmy villages				
Bitouga (2°07.133′N, 12°07.770′E)	21	13 (62)	0	–
Etho (2°05.728′N, 12°09.460′E)	28	9 (32)	0	–
Mixed villages				
Doumassi (2°09.212′N, 12°07.781′E)	32	13 (40.6)	5	1 (20)
Zangaville (2°04.474′N, 12°02.476′E)	13	5 (38.4)	10	6 (60)
Esseng (2°09.726′N, 12°08.321′E)	54	30 (55.5)	0	–
NkokAkom (2°10.900′N, 12°07.826′E)	11	8 (72.7)	12	5 (41.6)
Mimbang (2°11.593′N, 12°07.620′E)	11	7 (63.6)	11	8 (72.7)
Forestry villages				
TTIB (1°36. 201′N, 11°59. 986′E)	0	–	56	39 (69.6)
Bordamure (1°02.305′N, 11°41.629′E)	0	–	39	21 (53.8)
STIBG (1°00.472′N, 11°55.789′E)	0	–	39	31 (79.4)
Total	170	85 (50)	172	111 (64.5)

In each village, all volunteers were included in the study. Volunteers were recruited with their informed consent and in the case of children the consent of their parents. The translation of consent for participants was done by members of the NGO AGAFI (Gabonese Association of Assistance to Indigenous Women and Indigent). Before blood collection, a medical doctor examined each volunteer. Age and sex were retrieved for each patient. Blood was collected in EDTA tubes using venipuncture. Each sample was aliquoted and stored at −20 °C until used.

A total of 342 blood samples were obtained: 170 from Baka Pygmies (121 from MV and 49 from IPV) and 172 from Bantus (38 in MV and 134 in FV). This study was authorized by the Ministry of Health and the agreement of the National Ethics Committee on Research (authorization number: PROT No 0030/2013/SG/CNE).

### Determination of *Plasmodium falciparum* infections

For each blood sample, total DNA was extracted from 200 µl of blood using the DNeasy blood and tissue kit Qiagen (QIAamp^®^ DNA Blood Mini Kit) according to manufacturer’s instructions.

For each sample, *Plasmodium* infections were detected using the method based on the amplification of a portion of the *Plasmodium* cytochrome b (*cyt*-*b*) gene by a nested PCR as described in Additional file 1 [23]. The PCR amplified products were sequenced by Eurofins (MWG, France). Then, a logistic regression (binomial family) was used to test for variations of *P. falciparum* prevalence between Pygmies and Bantus (fixed effect) or between village types (fixed effect). In both cases, the factor ‘village of collection’ was considered as a random effect. Tests were realized using the R software [24] and the library lme 4 [25, 26].

### Molecular genotyping of *Plasmodium falciparum* isolates

Seven microsatellite markers as well as four candidate genes coding for antigenic proteins were genotyped.

The microsatellite markers amplified by semi-nested PCR are shown in Additional file 2. The PCR primer sets and amplification conditions were those described by [9, 27]. Fluorescence-labelled PCR products were sized on ABI Prism 310 genetic analyzer (Applied Biosystems), with a Genescan 500 LIZ internal size standard.

Regarding the candidate genes, merozoite surface protein 1 (*msp1*—block 2), merozoite surface protein 2 (*msp2*—block 3), glutamate-rich protein (*glurp*-region 2), and erythrocyte binding protein-175 (*eba*-*175*—region III) genes were genotyped as described in [21, 22, 28]. *Msp1* is a single copy gene located on chromosome 9. It encodes a merozoite surface antigen of approximately 190 kDa. The C-terminal part (MSP1_19_) is immunogenic and contains Epidermal Growth Factor (EGF)-like domains. MSP1_19_ is very rich in cysteine and is involved in the invasion of red blood cells by merozoites [29, 30]. MSP1 is composed of 17 blocks. Block 2, which is the first variable block, located at the N-terminal region is the most polymorphic. Block 2 of *msp1* was genotyped in this study. MSP-2 is located on chromosome 2 and encodes a 46–53 kDa antigenic protein located at the surface of the merozoite [31]. It consists of three regions: two semi-conserved (at the N-and C-terminal regions) and one polymorphic region composed of tandemly repetitive sequences [32–34]. The central polymorphic region (block 3) of *msp2* was genotyped in this study. The EBA-175 antigen is a molecule expressed at the surface of the parasite, which binds to red blood cells. It is involved in the invasion of the red blood cells [35–37]. It is a dimorphic molecule composed of two alleles: CAMP (C) and FCR3 (F). Dimorphism is related to two segments of region III. This is this region that has been genotyped in this study. Finally, GLURP is a 220 kDa protein expressed in both pre-erythrocytic and erythrocytic stages of *P. falciparum*, as well as on the surface of newly released merozoites in human host [38]. It consists of a N-terminal region of limited diversity named R0 followed by two polymorphic repeat regions named R1 and R2 [39]. The region 2 (R2) of GLURP has been genotyped in this study.

The specific primers used for genotyping each gene are given in Additional file 1. For *msp1* gene, three allelic families (K1, MAD20 and RO33) were determined. The two allelic families 3D7 and FC27 of the *msp2* gene were considered and the F (FCR3) and C (CAMP) alleles for the gene *eba*-*175*. For *glurp*, alleles were defined based on the amplicon size. PCR products were all visualized on a 1.5 % agarose gel electrophoresis.

### *Plasmodium falciparum* population genetic analyses

Allele frequency was computed as the proportion of the total of all alleles detected among the isolates examined (see Additional file 3).

All population genetic analyses were performed using FSTAT version 2.9.4 [40]. Genetic diversity within populations was assessed by the unbiased expected heterozygosity (*He*) [41]. Comparison of genetic diversity was performed either between Pygmy and Bantu populations or between IPVs against the others village types using a permutation test (1000 permutations).

Population genetic differentiation was estimated using the estimator (θ) of the Wright’s *F*-statistics *F*_ST_ [42, 43]. This index measures the genetic differentiation (i.e., the differences of allelic frequencies) between populations of interest. Deviation of θ from 0 was tested using a permutation test (1000 permutations). Mantel tests were used to test for patterns of isolation by distance (IBD). A partial Mantel test was performed to test for an effect of the ethnic group on *P. falciparum* genetic differentiation while controlling for geographic distance (1000 permutations). For this test, the first matrix was the matrix of *F*_ST_ computed between all pairs of populations. The second matrix was the matrix of pairwise geographic distances computed between populations and the third one was a matrix containing 1 when the populations belonged to the same ethnicity (Pygmy/Pygmy and Bantu/Bantu) and 2 when they belonged to different ethnic groups (Pygmy/Bantu).

An additional partial Mantel test was performed with two new groups of populations: one including only the IPVs that are strictly inhabited by Pygmies and the other including the other villages that are mostly inhabited by Bantus (MV + FV). MV isolates were indeed considered more likely to be representative of Bantus because, inside MVs, pygmies represent only a minority of individuals (even if they were sometimes sampled in majority) and also because no genetic differentiation was observed between *P. falciparum* populations collected from Pygmies and Bantus within these villages (see “Results”). For this test, the first matrix was the matrix of *F*_*ST*_ between populations, the second matrix corresponded to the geographic distances between populations and the third one contained 1 when the populations belonged to the same village type (IPV or MV + FV) and 2 when they belonged to two village types (IPV/MV + FV).

## Results

### Malaria prevalence and *Plasmodium* species

A total of 342 blood samples were analysed: 172 from Bantus and 170 from Pygmies. Distribution and origin of blood samples per village, village types (IPV, MV and FV) and ethnic groups are given in Table 1. A total of 196 samples were detected positive to *P. falciparum*. Prevalence varied from one village to another and is given in Table 1. Logistic regressions revealed no effect of the ethnic group (Pygmy vs Bantu, *P*-*value* = 0.154) on the prevalence of infection (while accounting for variations between villages) but differences between village types. Thus, although no significant differences were observed between IPVs and MVs (*P*-*value* = 0.49), a significant difference was observed between FVs and IPVs (*P*-*value* = 0.029) and between FVs and MVs (*P*-*value* = 0.041). Average infection prevalence was significantly higher in the villages from the forest concessions (FV) than in any other village types (IPV + MV).

### Microsatellite population structure

Seven microsatellite markers were genotyped in a total of 196 *P. falciparum* isolates from two IPVs, five Bantu/Pygmy: MVs, and from three villages of FVs representing 111 isolates from Bantus and 85 from Pygmies. All markers were highly polymorphic with a total number of alleles per locus observed varying from sixto 17. For each village type or ethnic group (Pygmy/Bantu), mean expected heterozygosity (*He*) is given in Table 2. No significant difference was observed between the estimates of genetic diversity (*He*) compared either between ethnic groups (Pygmies *vs* Bantus, *P*-*value* = 0.24) or village types (IPV *vs* MV + FV, *P*-*value* = 0.25).

**Table 2:** Mean genetic diversity (*He*) of *Plasmodium falciparum* populations estimated from the different village types and the different ethnic groups (All Pygmies and All Bantus) for microsatellite markers and antigen-coding genes

	Isolated pygmyvillages (IPV)	Mixed villages (MV)		Forestry villages (FV)	All pygmies	All bantus
		Pygmies	Bantus			
Microsatellites						
Ta60	0.835	0.817	0.828	0.789	0.826	0.808
Ara2	0.833	0.877	0.889	0.853	0.855	0.871
Taa87	0.819	0.847	0.916	0.832	0.833	0.874
Pfpk2	0.930	0.808	0.889	0.890	0.869	0.890
Polyα	0.944	0.899	0.932	0.891	0.922	0.912
Ta1	0.944	0.929	0.941	0.848	0.937	0.894
Taa81	0.814	0.834	0.792	0.835	0.824	0.814
Mean	0.874	0.859	0.884	0.848	0.866	0.866
Candidate genes						
MSP2	0.711	0.836	0.603	0.741	0.773	0.672
MSP1	0.821	0.718	0.829	0.724	0.769	0.776
EBA-175	0.220	0.361	0.423	0.384	0.291	0.404
GLURP	0.359	0.741	0.612	0.644	0.550	0.628
Mean	0.528	0.664	0.617	0.623	0.596	0.620

Values within each category correspond to the average of genetic diversity computed within each population

**Fig. 2:**
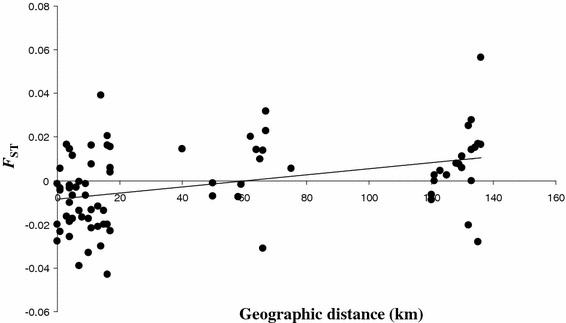
Relationship between pairwise geographic distances (in km) and pairwise *F*_ST_ estimates (θ) measured between each pair of populations, for microsatellite markers

Regarding genetic differentiation, the average *F*_ST_ (θ) computed over all loci and populations was low (θ = 0.005) and not significantly different from 0 (*P*-*value* = 0.102). Pairwise *F*_ST_ (θ) are given in Table 3. Within MVs with isolates from Pygmies and Bantus, no significant genetic differentiation was observed between the *P. falciparum* isolates collected from the different ethnic groups (Table 3). At a regional scale, a significant isolation by distance was observed among populations (Fig. 2, correlation coefficient r = 0.387; *P*-*value* = 0.002). Partial Mantel tests revealed no significant effect of the ethnic group or of the village type (Table 4).

**Table 3:** Pairwise *F*_ST_ estimates (θ) measured between each pair of populations

	Group	Village	Isolated Pygmy villages	Mixed villages								Forestry Villages		
			Pygmies	Pygmies	Pygmies	Pygmies	Bantus	Pygmies	Bantus	Pygmies	Bantus	Bantus	Bantus	Bantus
			Etho	Doumassi	Esseng	Mimbang	Mimbang	NkokAkom	NkokAkom	Zangaville	Zangaville	Bordamure	STIBG	TTIB
Microsatellites														
Isolated Pygmy villages	Pygmies	Bitouga	−0.0256	−0.0101	−0.0033	−0.0068	−0.0016	−0.0005	−0.039	0.0075	−0.0217	0.0075	0.0025	−0.0017
	Pygmies	Etho		−0.0138	−0.0168	0.0162	−0.0135	−0.0175	−0.033	−0.0117	−0.0212	0.008	0.0045	−0.0073
Mixed villages	Pygmies	Doumassi			−0.0069	−0.0175	0.0116	−0.0035	−0.0188	0.0392	−0.0301	0.0277	0.0058	0.02
	Pygmies	Esseng				−0.0022	0.0146	0.0164	−0.0166	−0.0202	−0.0138	0.0151	0.0113	0.0141
	Pygmies	Mimbang					*−0.0015*	0.0056	−0.0233	0.0057	0.0037	0.0164	0	0.0228
	Bantus	Mimbang						−0.0036	−0.0046	−0.0232	0.0156	0.0563	0.0142	0.0319
	Pygmies	NkokAkom							*−0.02*	0.0204	0.0163	0.0167	0.025	0.0139
	Bantus	NkokAkom								−0.0431	−0.0202	−0.0281	−0.0204	−0.0311
	Pygmies	Zangaville									*−0.0277*	0	−0.0065	−0.007
	Bantus	Zangaville										0.0025	−0.0087	−0.001
Forestry villages	Bantus	Bordamure											0.0144	0.0054
	Bantus	STIBG												0.01
Candidate genes														
Isolated Pygmy villages	Pygmies	Bitouga	−0.0417	0.0421	0.1063	0.1041	0.1398	0.0372	0.2664*	0.149	0.1301	0.1632	0.1509*	0.1428
	Pygmies	Etho		0.0027	0.0795	0.0244	0.1629	0.0272	0.2818	0.0324	0.0543	0.1335	0.1627*	0.1245
Mixed villages	Pygmies	Doumassi			0.0469	−0.0436	0.0255	−0.064	0.1047	−0.0423	−0.0001	0.0128	0.114*	0.0194
	Pygmies	Esseng				0.1123	0.1308	0.1079	0.2229*	−0.0417	0.0472	0.065	0.0752	0.1047*
	Pygmies	Mimbang					*0.0236*	−0.1455	0.0874	0.0718	−0.0164	0.0557	0.0987	0.0616
	Bantus	Mimbang						−0.0626	0.0374	0.0465	0.064	0.088	0.0498	0.0471
	Pygmies	NkokAkom							*0.0754*	0.1014	0.0076	0.0031	0.0273	0.0278
	Bantus	NkokAkom								0.2069	0.1619	0.1916	0.1838*	0.1617
	Pygmies	Zangaville									*−0.0312*	−0.0239	0.1373	−0.0645
	Bantus	Zangaville										0.0622	0.1055	0.0735
Forestry villages	Bantus	Bordamure											0.1085	0.0272
	Bantus	STIBG												0.1612*

Values in italics are *F*_ST_ values obtained within mixed villages between *P. falciparum* from Pygmies and Bantus

* *P*-*value* <0.05 (after Bonferroni correction)

**Table 4:** Partial correlation coefficients of partial Mantel tests realized for microsatellites (all loci) and each of the four candidate genes

	Distance	Within/between ethnies	Within/between village types
Microsatellites	0.387**	−0.145 ns	−0.15 ns
MSP2	0.258 ns	0.0156 ns	0.15 ns
MSP1	0.08 ns	−0.06 ns	−0.23 ns
EBA-175	0.025 ns	−0.05 ns	0.02 ns
GLURP	0.025 ns	0.09 ns	0.54**
All candidate genes	0.197 ns	0.11 ns	0.39**

*ns* non-significant

** *P*-*value* ≤0.01. *P*-*values* are given after Bonferroni corrections for multiple testing

### Candidate gene population structure

The genotyping of the different genes (*msp1*, *msp2*, *eba*-*175*, and *glurp*) was also performed using 196 *P. falciparum* isolates. For these genes, alleles were classified according to the size of the amplified fragment. The different alleles and their distribution in the different collection sites are presented in Additional file 3. Unbiased genetic diversity (*He*) estimated for each gene, each type of village and each population group is presented in Table 2. Comparison among ethnic groups revealed no difference between Pygmies and Bantus for any of these genes. The only significant difference was observed for *glurp* between IPV and the other villages (MV + FV, *P*-*value* = 0.018).

**Fig. 3:**
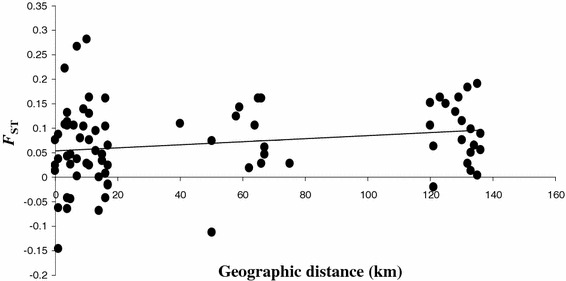
Relationship between pairwise geographic distances (in km) and pairwise *F*_ST_ estimates (θ) measured between each pair of populations, for antigenic markers

Regarding genetic differentiation, the average *F*_ST_ measured between populations over all loci was low (*F*_ST_ = 0.09) and significantly different from 0 (*P*-*value* ≤0.001). Within MVs, no significant genetic differentiation was observed between the isolates collected in Pygmies and Bantus (Table 3; Additional file 4). Although non-significant, the Mantel test revealed a similar regional pattern as the one observed for microsatellites, namely a global isolation by distance (r = 0.197; *P*-*value* = 0.093) (Fig. 3). Partial Mantel tests were realized for each gene. Results are given in Table 4. As shown, correlation coefficients for the ethnic group effect were non-significant. Regarding the village type effect (IPVs vs the others), all correlation coefficients were not significantly different from 0 except for GLURP for which a significant strong negative correlation coefficient was observed meaning that, for this gene, genetic differentiation was higher between village types (IPVs vs the others) than within.

## Discussion

In this study, the characterization of the genetic polymorphism of *P. falciparum* was under taken in isolated Baka Pygmy villages in Gabon and compared to neighbouring villages composed of mixed Bantu/Pygmy populations or villages of logging companies (only Bantus).

### *Plasmodium falciparum* infection prevalence

Over all samples (n = 342), around 57 % of the individuals were infected by *P. falciparum* (n = 196). No significant difference was observed in the prevalence of *P. falciparum* between Pygmies and Bantus. This result is at odds with various studies realized in the middle of the 20th Century in Central Africa, showing a global malaria infection rate lower in Pygmy camps than in surrounding Bantu villages. Explanations for these differences varied from one study to another. Schwetz attributed the differences to ethnic characteristics because they considered that the different human communities were submitted to a similar malaria risk [44]. However, some authors have noted a far lower number of malaria vectors in Pygmy camps (even in those temporarily sedentarized) than in neighbouring Bantu villages as well as differences in the composition of the vector communities [45, 46]. Thus, *Anopheles gambiae s.s*, the main vector of *Plasmodium* in Equatorial regions, was mostly absent from the undergrowth and so the Pygmy camps because of the ecological preferences of their larvae for temporarily sunned breeding sites [47].

The absence of differences observed in this study between the IPVs and the neighbouring Bantu villages (especially the MVs) can mainly be explained by the drastic cultural changes Pygmies have experienced in the last 50 years. Most Pygmies are no longer nomadic and now live in settlements very similar to those of the Bantus. Pygmy communities spend more time outside the forest in fixed settlements, within-village population densities have increased as well as their contact with the Bantus [20]. As a consequence, their settlements are now favourable to the development of *An. gambiae,* the main vector of *P. falciparum* and to an efficient malaria transmission. All these factors may explain the increase of malaria prevalence in present Pygmy communities and the similarity in the intensity of transmission with the neighbouring Bantu communities.

### *Plasmodium falciparum* population genetic structure: microsatellites

Because microsatellite markers are expected to be neutral, population genetic structure estimated using these markers is considered to be mainly the consequence of demographic events and not selection acting on these populations. Regarding *P. falciparum* population genetic diversity and its distribution, no substantial difference between village types or ethnic groups (Pygmies vs. Bantus) was observed. Genetic diversity as measured by expected heterozygosity was high in the different population groups and equivalent to levels of diversity measured in other African areas with a hyper endemic transmission [27, 48] (as is the case for Gabon).

Levels of genetic differentiation measured between villages were small and not significantly higher between populations belonging to different village types (IPV, MV and FV) or ethnic groups. No significant genetic differentiation was observed between Pygmies and Bantus within MVs. Over all the studied area, a pattern of isolation by distance was observed (genetic differentiation is on average lower for populations that are geographically close) thus suggesting that exchanges of parasites among villages occur mostly at small geographic distances and that no particular barrier to migration exists between Pygmy and Bantu communities (Fig. 2).

### *Plasmodium falciparum* population genetic structure: candidate genes

The distribution of genetic diversity at candidate genes, if under selection, is the consequence of the demographic history of the populations (as for microsatellites) but also of the action of selection. Comparing their population structure to that of microsatellites is thus a way to detect phenomenon of selection and in particular phenomenon of local adaptation for certain genes.

Overall, similar patterns of population structure were observed at the candidate genes, namely no differences in the levels of genetic diversity among village types or ethnic groups and a global isolation by distance at the scale of the study (although only marginally significant). The only substantial difference was observed for the gene GLURP. Not only the level of genetic diversity of this gene was significantly lower in the IPVs (Table 2) than in the other village types but also the level of genetic differentiation was significantly more important between village types (IPV against MV and FV) than inside, everything else being equal (geographical distance). This suggests therefore the existence of a potential phenomenon of disruptive selection acting on this gene between the IPVs and the other villages.

GLURP is expressed at different stages in the parasite including mosquito and vertebrate stages [49]. If disruptive selection indeed occurs at this gene, this could then be due to differential effects of the Pygmy and Bantu immune systems or to the mosquito vectors that are involved in transmission in the different village types. If ethnicity is the selective factor, one could however wonder why no significant genetic differences were observed within mixed villages between isolates collected from Pygmies or Bantus. This could be due to a lack of statistical power (due to small sample sizes) or to the strong parasite migration occurring between ethnic groups within village, a phenomenon known to prevent the evolution of local adaptation [50].

The findings of this are compatible with results of another study comparing the level of population structure among *P. falciparum* populations collected in different regions of the world at genes encoding ten leading vaccine antigens [51], including the four candidate genes studied here. In this study, the strongest level of genetic differentiation among different populations was also observed at the *glurp* gene. Authors attributed this observation to several possible factors, including variations in human genetic resistance to malaria or adaptation to different anopheline species transmitting *P. falciparum* worldwide [51].

## Conclusion

Very few studies have been conducted on the population genetic structure of *P. falciparum* in Pygmies. In this study, the prevalence and genetic diversity of *P. falciparum* observed in Baka demonstrates a significant transmission of the parasite in this population, and some exchanges of parasites with the Bantu neighbours. Despite that, some antigen-coding genes seem to have had a particular evolutionary trajectory in certain Pygmy populations due to specific local human and/or mosquito characteristics. It is necessary to extend this research to other sites, including the Bakoya Pygmies in the northeast and Babongo Pygmies in southern Gabon, as well as to other sites in Central Africa. It would in particular be interesting to analyse the prevalence and population genetic structure in the last Pygmy groups that have conserved a traditional nomadic and forest-dependent way of life, such as in the Central African Republic.

## Additional files

Additional file 1: Sequences of primers used for genotyping *P. falciparum* genes. This table shows the primers sequences (cytochrome b, MSP1, MSP2, EBA 175 and GLURP) used. Additional file 2: Microsatellite loci characteristics for *P. falciparum.* Presentation of the seven microsatellite sequences used and their repeat motif. Additional file 3: Allelic diversity of MSP-1, MSP-2, EBA-175 and Glurp of *P. falciparum* in Bantus and Baka Pygmies. Distribution of alleles of genes MSP1, MSP2, EBA 175 and GLUPR by Population Group. Additional file 4: Pairwise *F*_ST_ estimates (θ) measured between each pair of populations for each of the four candidate genes (MSP2, MSP1, EBA-175, GLURP). Values in bold are *F*_ST_ values obtained within mixed villages between *P. falciparum* from Pygmies and Bantus. **P*-*value* < 0.05 (after Bonferroni correction). NA: not available. Pairwise *F*_ST_ estimates (θ). Values in bold are *F*_ST_ values obtained within mixed villages between *P. falciparum* from Pygmies and Bantus. **P*-*value* < 0.05 (after Bonferroni correction). NA: not available.

## References

[1] WHO (2013). World Malaria Report 2013.

[2] Day KP, Marsh K (1991). Naturally acquired immunity to *Plasmodium falciparum*. Immunol Today.

[3] Mwingira F, Nkwengulila G, Schoepflin S, Sumari D, Beck HP, Snounou G (2011). *Plasmodium falciparum* msp1, msp2 and glurp allele frequency and diversity in sub-Saharan Africa. Malar J.

[4] Healer J, Murphy V, Hodder AN, Masciantonio R, Gemmill AW, Anders RF (2004). Allelic polymorphisms in apical membrane antigen-1 are responsible for evasion of antibody-mediated inhibition in *Plasmodium falciparum*. Mol Microbiol.

[5] Manske M, Miotto O, Campino S, Auburn S, Almagro-Garcia J, Maslen G (2012). Analysis of *Plasmodium falciparum* diversity in natural infections by deep sequencing. Nature.

[6] Prugnolle F, Durand P, Jacob K, Razakandrainibe F, Arnathau C, Villarreal D (2008). A comparison of *Anopheles gambiae* and *Plasmodium falciparum* genetic structure over space and time. Microbes Infect.

[7] Larranaga N, Mejia RE, Hormaza JI, Montoya A, Soto A, Fontecha GA (2013). Genetic structure of *Plasmodium falciparum* populations across the Honduras-Nicaragua border. Malar J.

[8] Annan Z, Durand P, Ayala FJ, Arnathau C, Awono-Ambene P, Simard F (2007). Population genetic structure of *Plasmodium falciparum* in the two main African vectors, Anopheles gambiae and Anopheles funestus. Proc Natl Acad Sci USA.

[9] Anderson TJ, Su XZ, Bockarie M, Lagog M, Day KP (1999). Twelve microsatellite markers for characterization of *Plasmodium falciparum* from finger-prick blood samples. Parasitology.

[10] Yalcindag E, Elguero E, Arnathau C, Durand P, Akiana J, Anderson TJ (2012). Multiple independent introductions of *Plasmodium falciparum* in South America. Proc Natl Acad Sci USA.

[11] Mobegi VA, Loua KM, Ahouidi AD, Satoguina J, Nwakanma DC, Amambua-Ngwa A (2012). Population genetic structure of *Plasmodium falciparum* across a region of diverse endemicity in West Africa. Malar J.

[12] Aubouy A, Migot-Nabias F, Deloron P (2003). Polymorphism in two merozoite surface proteins of *Plasmodium falciparum* isolates from Gabon. Malar J.

[13] Aubouy A, Migot-Nabias F, Deloron P (2007). Correlations between treatment outcome and both anti-MSP119 antibody response and erythrocyte-related genetic factors in *Plasmodium falciparum* malaria. Infect Genet Evol.

[14] Peyerl-Hoffmann G, Jelinek T, Kilian A, Kabagambe G, Metzger WG, von Sonnenburg F (2001). Genetic diversity of *Plasmodium falciparum* and its relationship to parasite density in an area with different malaria endemicities in West Uganda. Trop Med Int Health.

[15] Soulama I, Nebie I, Ouedraogo A, Gansane A, Diarra A, Tiono AB (2009). *Plasmodium falciparum* genotypes diversity in symptomatic malaria of children living in an urban and a rural setting in Burkina Faso. Malar J.

[16] Olasehinde GI, Yah CS, Singh R, Ojuronbge OO, Ajayi AA, Valecha N (2012). Genetic diversity of *Plasmodium falciparum* field isolates from south western Nigeria. Afr Health Sci.

[17] Quintana-Murci L, Quach H, Harmant C, Luca F, Massonnet B, Patin E (2008). Maternal traces of deep common ancestry and asymmetric gene flow between Pygmy hunter-gatherers and Bantu-speaking farmers. Proc Natl Acad Sci USA.

[18] Verdu P, Austerlitz F, Estoup A, Vitalis R, Georges M, Thery S (2009). Origins and genetic diversity of pygmy hunter-gatherers from Western Central Africa. Curr Biol.

[19] Ohenjo N, Willis R, Jackson D, Nettleton C, Good K, Mugarura B (2006). Health of Indigenous people in Africa. Lancet.

[20] Stephens C, Porter J, Nettleton C, Willis R (2006). Disappearing, displaced, and undervalued: a call to action for Indigenous health worldwide. Lancet.

[21] Ranford-Cartwright LC, Taylor J, Umasunthar T, Taylor LH, Babiker HA, Lell B (1997). Molecular analysis of recrudescent parasites in a *Plasmodium falciparum* drug efficacy trial in Gabon. Trans R Soc Trop Med Hyg.

[22] Ntoumi F, Ngoundou-Landji J, Lekoulou F, Luty A, Deloron P, Ringwald P (2000). Site-based study on polymorphism of *Plasmodium falciparum* MSP-1 and MSP-2 genes in isolates from two villages in Central Africa. Parassitologia.

[23] Prugnolle F, Durand P, Neel C, Ollomo B, Ayala FJ, Arnathau C (2010). African great apes are natural hosts of multiple related malaria species, including *Plasmodium falciparum*. Proc Natl Acad Sci USA.

[24] R-Development-Core-Team. R: a language and environment for statistical computing. In R Foundation for Statistical Computing, Vienna, Austria http://www.R-projetorg/, 2014.

[25] Doran H, Bates D, Bliese P, Dowling M (2007). Estimating the multilevel Rasch Model: with the lme4 package. J Stat Softw.

[26] Bates D, Maechler M, Bolker B. lme4: Linear Mixed-Effects Models Using S4 Classes. R package version 0.999375-38. http://CRAN.R-projectorg/package=lme4, 2011.

[27] Leclerc MC, Durand P, de Meeus T, Robert V, Renaud F (2002). Genetic diversity and population structure of *Plasmodium falciparum* isolates from Dakar, Senegal, investigated from microsatellite and antigen determinant loci. Microbes Infect.

[28] Toure FS, Mavoungou E, Ndong JM, Tshipamba P, Deloron P (2001). Erythrocyte binding antigen (EBA-175) of *Plasmodium falciparum*: improved genotype determination by nested polymerase chain reaction. Trop Med Int Health.

[29] Blackman MJ, Heidrich HG, Donachie S, McBride JS, Holder AA (1990). A single fragment of a malaria merozoite surface protein remains on the parasite during red cell invasion and is the target of invasion-inhibiting antibodies. J Exp Med.

[30] Holder AA, Guevara Patino JA, Uthaipibull C, Syed SE, Ling IT, Scott-Finnigan T (1999). Merozoite surface protein 1, immune evasion, and vaccines against asexual blood stage malaria. Parassitologia.

[31] Clark JT, Donachie S, Anand R, Wilson CF, Heidrich HG, McBride JS (1989). 46-53 kilodalton glycoprotein from the surface of *Plasmodium falciparum* merozoites. Mol Biochem Parasitol.

[32] Smythe JA, Coppel RL, Day KP, Martin RK, Oduola AM, Kemp DJ (1991). Structural diversity in the *Plasmodium falciparum* merozoite surface antigen 2. Proc Natl Acad Sci USA.

[33] Fenton B, Clark JT, Khan CM, Robinson JV, Walliker D, Ridley R (1991). Structural and antigenic polymorphism of the 35- to 48-kilodalton merozoite surface antigen (MSA-2) of the malaria parasite *Plasmodium falciparum*. Mol Cell Biol.

[34] Irion A, Beck HP, Felger I (1997). New repeat unit and hot spot of recombination in FC27-type alleles of the gene coding for *Plasmodium falciparum* merozoite surface protein 2. Mol Biochem Parasitol.

[35] Sim BK, Chitnis CE, Wasniowska K, Hadley TJ, Miller LH (1994). Receptor and ligand domains for invasion of erythrocytes by *Plasmodium falciparum*. Science.

[36] Klotz FW, Orlandi PA, Reuter G, Cohen SJ, Haynes JD, Schauer R (1992). Binding of *Plasmodium falciparum* 175-kilodalton erythrocyte binding antigen and invasion of murine erythrocytes requires N-acetylneuraminic acid but not its O-acetylated form. Mol Biochem Parasitol.

[37] Orlandi PA, Klotz FW, Haynes JD (1992). A malaria invasion receptor, the 175-kilodalton erythrocyte binding antigen of *Plasmodium falciparum* recognizes the terminal Neu5Ac(alpha 2-3)Gal- sequences of glycophorin A. J Cell Biol.

[38] Borre MB, Dziegiel M, Hogh B, Petersen E, Rieneck K, Riley E (1991). Primary structure and localization of a conserved immunogenic *Plasmodium falciparum* glutamate rich protein (GLURP) expressed in both the preerythrocytic and erythrocytic stages of the vertebrate life cycle. Mol Biochem Parasitol.

[39] de Stricker K, Vuust J, Jepsen S, Oeuvray C, Theisen M (2000). Conservation and heterogeneity of the glutamate-rich protein (GLURP) among field isolates and laboratory lines of *Plasmodium falciparum*. Mol Biochem Parasitol.

[40] Goudet J. FSTAT version 2.9.4: a programm to estimation genetics parameters. Updated from Goudet (1995). Available from http://www.unilch/izea/softwares/fstathtm 2003.

[41] Nei M (1967). Modification of linkage intensity by natural selection. Genetics.

[42] Wright S (1951). The genetical structure of populations. Ann Eugen.

[43] Weir BS, Cockerham CC (1984). Estimating F-statistic for the analysis of population structure. Evolution.

[44] Schwetz J, Baumann H, Peel A, Droeshaut F (1933). Etude comparative de la malaria chez les Pygmées et les indigènes ordinaires de la forêt de l’Ituri (Congo Belge). Bull Soc Path Exot.

[45] Languillon J, Mouchet J (1955). Résultats des enquêtes épidémiologiques et entomologiques sur le paludisme dans les régions de Lom et Kadei et du Boumba-Ngoko.

[46] Carnevale P, Rickenbach A, Frezil JL (1978). Les maladies transmises et leurs vecteurs chez les Pygmées Aka de Basse Lobaye.

[47] Mouchet J, Carnevale P, Coosemans M, Fontenille D, Ravaon-Jaahary C, Richard A (1993). Typologie du paludisme en Afrique. Cahiers de santé.

[48] Bogreau H, Renaud F, Bouchiba H, Durand P, Assi SB, Henry MC (2006). Genetic diversity and structure of African *Plasmodium falciparum* populations in urban and rural areas. Am J Trop Med Hyg.

[49] Lopez-Barragan MJ, Lemieux J, Quinones M, Williamson KC, Molina-Cruz A, Cui K (2011). Directional gene expression and antisense transcripts in sexual and asexual stages of *Plasmodium falciparum*. BMC Genom.

[50] Lenormand T (2002). Gene flow and the limits to natural selection. Trends Ecol Evol.

[51] Barry AE, Schultz L, Buckee CO, Reeder JC (2009). Contrasting population structures of the genes encoding ten leading vaccine-candidate antigens of the human malaria parasite, *Plasmodium falciparum*. PLoS One.

