# Supplementary material for: Genetic diversity of Plasmodium falciparum isolates from Baka Pygmies and their Bantu neighbours in the north of Gabon
Source: Malar J. 2015 Oct 9;14:395. doi: 10.1186/s12936-015-0862-5 (PMC4599724; doi:10.1186/s12936-015-0862-5)
Supplement: Additional file 1: — Sequences of primers used for genotyping P. falciparum genes. This table shows the primers sequences (cytochrome b, MSP1, MSP2, EBA 175 and GLURP) used. [file 12936_2015_862_MOESM1_ESM.docx]

**Additional file 1 Sequences of primers used for genotyping *P. falciparum* genes**

| **Locus** | **Amplification** | **Primers** |  | **Sequences** |
| --- | --- | --- | --- | --- |
| **Cytochrome b** | Primary  Secondary | DW2/F  DW4/R  CYTb1/F  CYTb2/R |  | 5’-TAATGCCTAGACGTATTCCTAGTTATCCAG-3’  5’-TGTTTGCTTGGGAGCTGTAATCATAATGTG-3’  5’-CTCTATTATTTAGTTAAAGCACA-3’  5’-ACAGAATAATCTTAGCACC-3’ |
| **MSP1** | Primary  Secondary | A  B  K1/A  K1/B  Ro33/A  Ro33/B  Mad20/A  Mad20/A |  | 5’-AAGCTTTAGAAGATGCAGTATTGAC-3’  5’-ATTCATTAATTTCTTCATATCCATC-3  5’-AAGAAATTACTACAAAAGGTG-3’  5’-TGCATCAGCTGGAGGGCTTGCACCAGA-3’  5’-AGGATTTGCAGCACCTGGAGATCT-3’  5’-GAGCAAATACTCAAGTTGTTGCA-3’  5’-TGAATTATCTGAAGGATTTGTACGTCT-3’  5’-GAACAAGTCGAACAGCTGTTA-3’ |
| **MSP2** | Primary  Secondary | 1  4  FC27/A  FC27/B  3D7/A  3D7/B |  | 5’-ATGAAGGTAATTAAAACATTGTCTATTATA-3’  5’-ATATGGAAAAGATAAAACAAGTGTTGCGT-3’  5’-GCAAATGAAGGTTCTAATACTAATAG-3’  5’-GCTTTGGGTCCTTCTTCAGTTGATTC-3’  5’-GCAGAAAGTAAGCCTTCTACTGGTGCT-3’  5’-GATTTGTTTCGGCATTATTATGA-3’ |
| **EBA 175** | Primary  Secondary | EBA1  EBA2  EBA3  EBA4 |  | 5’-CAAGAAGCAGTTCCTGAGGAA-3’  5’-TCTCAACATTCATATTAACAATTC-3’  5’-GAGGAAAACACTGAAATAGCACAC-3’  5’-CAATTCCTCCAGACTGTTGAACAT-3’ |
| **GLURP** | Primary  Secondary | Glurp A/F  Glurp A/R  Glurp B/F  Glurp B/R |  | 5’-ACATGCAAGTGTTGATCC-3’  5’-GATGGTTTGGGAGTAACG-3’  5’-TGAATTCGAAGATGTTCACACTGAAC-3’  5’-TGTAGGTACCACGGGTTCTTGTGG-3’ |
