# Supplementary material for: Genetic diversity of Plasmodium falciparum isolates from Baka Pygmies and their Bantu neighbours in the north of Gabon
Source: Malar J. 2015 Oct 9;14:395. doi: 10.1186/s12936-015-0862-5 (PMC4599724; doi:10.1186/s12936-015-0862-5)
Supplement: Additional file 2: — Microsatellite loci characteristics for P. falciparum. Presentation of the seven microsatellite sequences used and their repeat motif. [file 12936_2015_862_MOESM2_ESM.docx]

**Additional file 2 Microsatellite loci characteristics for *P. falciparum***

| **Locus** | **Code** | **Chromosome**  **location** | **Repeat**  **motif** | **GeneBank Accession**  **number** | **Primers sequence (5’-3’)** |
| --- | --- | --- | --- | --- | --- |
| **Ta1** | P18 | Chr. 6 | (TAA) (TGA) | AF010507 | F1: CTACATGCCTAATGAGCA  R: TTTTATCTTCATCCCCAC  F2: CCGTCATAAGTGCAGAGC |
| **Poly α** | P20 | Chr. 4 | (TAA) | L18875 | R1: ATCAGATAATTGTTGGTA  F: AAAATATAGACGAACAGA  R2: GAAATTATAACTCTACCA |
| **Ta60** | P21 | Chr. 13 | (TAA) | AF010556 | F1: CTCAAAGAAAAATAATTCA  R: AAAAAGGAGGATAAATACAT  F2: TAGTAACGATGTTGACAA |
| **Ara2** | P22 | Chr. 11 | (TAA) | X17484 | F1: GTACATATGAATCACCAA  R: GCTTTGAGTATTATTAATA  F2: GAATAAACAAAGTATTGCT |
| **Pfpk2** | P24 | Chr. 12 | (ATT) | X63648 | R1: CCTCAGACTGAAATGCAT  F: CTTTCATCGATACTACGA  R2: AAAGAAGGAACAAGCAGA |
| **Taa87** | P25 | Chr. 6 | (CAA) (TAA) | AF010571 | F1: ATGGGTTAAATGACGTACA  R: ACATGTTCATATTACTCAC  F2: AATGGCAACACCATTCAAC |
| **Taa81** | P27 | Chr. 5 | TAA | AF010510 | F1: GAAGAAATAAGGGAAGGT  R: TTTCACACAACACAGGATT  F2: TGGACAAATGGGAAAGGATA |
