# Supplementary material for: Genetic diversity of Plasmodium falciparum isolates from Baka Pygmies and their Bantu neighbours in the north of Gabon
Source: Malar J. 2015 Oct 9;14:395. doi: 10.1186/s12936-015-0862-5 (PMC4599724; doi:10.1186/s12936-015-0862-5)
Supplement: Additional file 3: — Allelic diversity of MSP-1, MSP-2, EBA-175 and Glurp of P. falciparum in Bantus and Baka Pygmies. Distribution of alleles of genes MSP1, MSP2, EBA 175 and GLUPR by Population Group. [file 12936_2015_862_MOESM3_ESM.docx]

**Additional file 3 Allelic diversity of MSP-1, MSP-2, EBA-175 and GLURP of *P. falciparum* in Bantus and Baka Pygmies**

| **Locus** | **Allele**  **size (bp)** | **Isolated Pygmy Villages** | |  | **Mixed Villages** | |  | **Forestry Villages** | |
| --- | --- | --- | --- | --- | --- | --- | --- | --- | --- |
|  |  | **N** | **Allele (%)** |  | **N** | **Allele (%)** |  | **N** | **Allele (%)** |
| **MSP1**  K1-a  K1-b  K1-c  K1-d  K1-e  K1-f  K1-g  Mad20-a  Mad20-b  Mad20-c  Mad20-d  Mad20-e  Ro33-a  Ro33-b  **Total fragment** | 150  180  200  220  250  280  300  150  180  200  230  250  150  200 | 0  5  1  2  3  0  0  0  0  5  0  0  7  0  **23** | (21.7)  (4.3)  (8.7)  (13)  (21.7)  (30.4) |  | 1  5  24  13  1  1  6  6  2  14  7  0  22  0  **102** | (0.9)  (4.9)  (23.5)  (12.7)  (0.9)  (0.9)  (5.8)  (5.8)  (1.9)  (13.7)  (6.8)  -  (21.6) |  | 2  5  30  16  1  3  1  0  3  12  6  5  35  2  **121** | (1.6)  (4.1)  (24.8)  (13.2)  (0.8)  (2.5)  (0.8)  -  (2.5)  (9.9)  (4.9)  (4.1)  (28.9)  (1.6) |
| **MSP2**  FC27-a  FC27-b  FC27-c  FC27-d  FC27-e  3D7-a  3D7-b  3D7-c  3D7-d  3D7-e  3D7-f  **Total fragment** | 300  350  420  500  600  270  300  390  400  500  600 | 2  9  5  0  0  4  0  0  0  0  0  **20** | (10)  (45)  (25)  (20) |  | 2  14  18  0  0  9  4  1  3  14  1  **66** | (3)  (21.2)  (27.3)  (13.6)  (6.1)  (1.5)  (4.5)  (21.2)  (1.5) |  | 0  0  14  13  3  2  10  9  0  0  0  **51** | (27.4)  (25.5)  (5.8)  (3.9)  (19.6)  (17.6) |
| **GLURP**  G1  G2  G3  G4  **Total fragment** | 400  600  1000  1300 | 5  0  0  19  **24** | (20.8)  (79.2) |  | 28  16  29  32  **105** | (26.6)  (15.2)  (27.6)  (30.5) |  | 42  20  23  32  **117** | (35.9)  (17.1)  (19.6)  (27.4) |
| **EBA-175**  F  C  **Total fragment** | 795  714 | 18  2  **20** | (90)  (10) |  | 70  32  **102** | (68.6  (31.4 |  | 69  29  **98** | (70.4)  (29.6) |
