# Supplementary material for: Genetic diversity of Plasmodium falciparum isolates from Baka Pygmies and their Bantu neighbours in the north of Gabon
Source: Malar J. 2015 Oct 9;14:395. doi: 10.1186/s12936-015-0862-5 (PMC4599724; doi:10.1186/s12936-015-0862-5)
Supplement: Additional file 4: — Pairwise F ST estimates (θ) measured between each pair of populations for each of the four candidate genes (MSP2, MSP1, EBA-175, GLURP). Values in bold are F ST values obtained within mixed villages between P. falciparum from Pygmies and Bantus. *P-value < 0.05 (after Bonferroni correction). NA: not available. Pairwise F ST estimates (θ). Values in bold are F ST values obtained within mixed villages between P. falciparum from Pygmies and Bantus. *P-value < 0.05 (after Bonferroni correction). NA: not available. [file 12936_2015_862_MOESM4_ESM.docx]

**Additional file 4 Pairwise *F*_ST_ estimates (θ) measured between each pair of populations for each of the four candidate genes (MSP2, MSP1, EBA-175, GLURP). Values in bold are *F*_ST_ values obtained within mixed villages between *P. falciparum* from Pygmies and Bantus. **P-value*<0.05 (after Bonferroni correction). NA: not available.**

|  | **Ethny** |  | **Pygmies** | **Pygmies** | **Pygmies** | **Pygmies** | **Bantus** | **Pygmies** | **Bantus** | **Pygmies** | **Bantus** | **Bantus** | **Bantus** | **Bantus** |
| --- | --- | --- | --- | --- | --- | --- | --- | --- | --- | --- | --- | --- | --- | --- |
| **MSP2**  **Isolated Pygmy Villages** |  | **Village** | **Etho** | **Doumassi** | **Esseng** | **Mimbang** | **Mimbang** | **Nkok Akom** | **Nkok Akom** | **Zangaville** | **Zangaville** | **Bordamure** | **STIBG** | **TTIB** |
|  | Pygmies | Bitouga | 0.0095 | -0.1221 | 0.128 | -0.0119 | 0.1 | -0.0413 | 0.0811 | NA | 0.102 | 0.1318 | 0.21* | 0.1224 |
|  | Pygmies | Etho |  | -0.0669 | 0.1768 | -0.14 | 0.3247 | 0.04 | 0.3016 | NA | 0.0368 | 0.2074 | 0.3181* | 0.2309 |
| **Mixed Villages** | Pygmies | Doumassi |  |  | 0.1 | -0.0937 | 0.1096 | -0.0957 | 0.0954 | NA | 0.0464 | 0.1043 | 0.1992 | 0.1075 |
|  | Pygmies | Esseng |  |  |  | 0.1635 | 0.2763 | 0.1529 | 0.2816 | NA | 0.074 | 0.0874 | 0.0707 | 0.2321* |
|  | Pygmies | Mimbang |  |  |  |  | **0.2055** | -0.2135 | 0.1992 | NA | -0.053 | 0.1169 | 0.2492* | 0.0957 |
|  | Bantus | Mimbang |  |  |  |  |  | -0.1208 | 0.0933 | NA | 0.2461 | 0.1368 | 0.1692 | 0.074 |
|  | Pygmies | Nkok Akom |  |  |  |  |  |  | **0.1176** | NA | -0.0158 | -0.0146 | 0.0652 | -0.0456 |
|  | Bantus | Nkok Akom |  |  |  |  |  |  |  | NA | 0.2617 | 0.2261 | 0.3271 | 0.0955 |
|  | Pygmies | Zangaville |  |  |  |  |  |  |  |  | **NA** | NA | NA | NA |
|  | Bantus | Zangaville |  |  |  |  |  |  |  |  |  | 0.081 | 0.1499* | 0.1803* |
| **Forestry Villages** | Bantus | Bordamure |  |  |  |  |  |  |  |  |  |  | 0.0692 | 0.0688 |
|  | Bantus | STIBG |  |  |  |  |  |  |  |  |  |  |  | 0.2247* |
|  |  |  |  |  |  |  |  |  |  |  |  |  |  |  |
| **MSP1** |  |  | **Etho** | **Doumassi** | **Esseng** | **Mimbang** | **Mimbang** | **Nkok Akom** | **Nkok Akom** | **Zangaville** | **Zangaville** | **Bordamure** | **STIBG** | **TTIB** |
| **Isolated Pygmy Villages** | Pygmies | Bitouga | -0.0756 | -0.039 | 0.0456 | 0.0232 | -0.0223 | -0.0283 | -0.0666 | -0.0515 | 0.0567 | 0.0151 | -0.0011 | -0.0369 |
|  | Pygmies | Etho |  | -0.0297 | 0.0238 | 0.0262 | -0.0066 | -0.0155 | -0.0526 | -0.0663 | 0.0407 | 0.0076 | -0.0159 | -0.028 |
| **Mixed Villages** | Pygmies | Doumassi |  |  | 0.0267 | 0.0379 | 0.0073 | -0.0307 | -0.0297 | 0.0122 | 0.0466 | -0.0259 | 0.0226 | -0.0313 |
|  | Pygmies | Esseng |  |  |  | 0.1494 | 0.1529 | 0.1318 | 0.0641 | -0.0783 | 0.1223 | -0.013 | 0.0873 | 0.0296 |
|  | Pygmies | Mimbang |  |  |  |  | **-0.074** | -0.1113 | -0.0398 | 0.1539 | 0.0418 | 0.0969 | -0.0205 | 0.043 |
|  | Bantus | Mimbang |  |  |  |  |  | -0.1226 | -0.0708 | 0.1304 | 0.0178 | 0.0922 | -0.0248 | 0.0131 |
|  | Pygmies | Nkok Akom |  |  |  |  |  |  | **-0.0903** | 0.1579 | 0.0609 | 0.0511 | -0.0546 | -0.0104 |
|  | Bantus | Nkok Akom |  |  |  |  |  |  |  | 0.0066 | 0.0407 | 0.0204 | -0.0266 | -0.0365 |
|  | Pygmies | Zangaville |  |  |  |  |  |  |  |  | **0.0676** | -0.0074 | 0.0808 | -0.0101 |
|  | Bantus | Zangaville |  |  |  |  |  |  |  |  |  | 0.1114 | 0.0511 | 0.0798 |
| **Forestry Villages** | Bantus | Bordamure |  |  |  |  |  |  |  |  |  |  | 0.0574 | -0.0032 |
|  | Bantus | STIBG |  |  |  |  |  |  |  |  |  |  |  | 0.025 |

**Additional file 4**

| **Village type** |  |  | **Isolated Pygmy Villages** | **Mixed villages** | | | | | | | | | | | | | | | **Forestry Villages** | | |
| --- | --- | --- | --- | --- | --- | --- | --- | --- | --- | --- | --- | --- | --- | --- | --- | --- | --- | --- | --- | --- | --- |
|  | **Ethny** |  | **Pygmies** | **Pygmies** | | **Pygmies** | | **Pygmies** | | **Bantus** | | **Pygmies** | | **Bantus** | | **Pygmies** | | **Bantus** | **Bantus** | **Bantus** | **Bantus** |
| **EBA-175** |  | **Villages** | **Etho** | **Doumassi** | **Esseng** | | **Mimbang** | | **Mimbang** | | **Nkok Akom** | | **Nkok Akom** | | **Zangaville** | | **Zangaville** | | **Bordamure** | **STIBG** | **TTIB** |
| **Isolated Pygmy Villages** | Pygmies | Bitouga | -0.0964 | 0.0494 | 0.1496 | | -0.0964 | | 0.1154 | | -0.1304 | | -0.0974 | | 0.336 | | 0.1944 | | 0.036 | -0.0347 | 0.2291 |
|  | Pygmies | Etho |  | -0.0781 | 0.0389 | | -0.1667 | | -0.0263 | | -0.098 | | -0.153 | | 0.125 | | 0.0299 | | -0.0652 | -0.0938 | 0.1131 |
| **Mixed Villages** | Pygmies | Doumassi |  |  | -0.0338 | | -0.0781 | | -0.086 | | 0.0486 | | -0.0496 | | -0.0241 | | -0.0659 | | -0.0695 | -0.0024 | 0.0136 |
|  | Pygmies | Esseng |  |  |  | | 0.0389 | | -0.0514 | | 0.1564 | | 0.0667 | | -0.0714 | | -0.0652 | | 0.0053 | 0.11 | -0.0118 |
|  | Pygmies | Mimbang |  |  |  | |  | | **-0.0263** | | -0.098 | | -0.153 | | 0.125 | | 0.0299 | | -0.0652 | -0.0938 | 0.1131 |
|  | Bantus | Mimbang |  |  |  | |  | |  | | 0.1111 | | 0.0061 | | -0.0772 | | -0.0904 | | -0.0482 | 0.0579 | -0.0224 |
|  | Pygmies | Nkok Akom |  |  |  | |  | |  | |  | | **-0.1089** | | 0.3143 | | 0.1837 | | 0.0336 | -0.054 | 0.2382 |
|  | Bantus | Nkok Akom |  |  |  | |  | |  | |  | |  | | 0.1732 | | 0.0674 | | -0.0414 | -0.0817 | 0.1422 |
|  | Pygmies | Zangaville |  |  |  | |  | |  | |  | |  | |  | | **-0.1304** | | 0.04 | 0.2494 | -0.1029 |
|  | Bantus | Zangaville |  |  |  | |  | |  | |  | |  | |  | |  | | -0.019 | 0.1292 | -0.0587 |
| **Forestry Villages** | Bantus | Bordamure |  |  |  | |  | |  | |  | |  | |  | |  | |  | 0.0021 | 0.0588 |
|  | Bantus | STIBG |  |  |  | |  | |  | |  | |  | |  | |  | |  |  | 0.1899 |
|  |  |  |  |  |  | |  | |  | |  | |  | |  | |  | |  |  |  |
| **GLURP** |  |  | **Etho** | **Doumassi** | **Esseng** | | **Mimbang** | | **Mimbang** | | **Nkok Akom** | | **Nkok Akom** | | **Zangaville** | | **Zangaville** | | **Bordamure** | **STIBG** | **TTIB** |
| **Isolated Pygmy Villages** | Pygmies | Bitouga | -0.0458 | 0.2837 | 0.1113 | | 0.351 | | 0.3519* | | 0.257 | | 0.7166* | | 0.2717 | | 0.2356 | | 0.3815* | 0.2851* | 0.2705 |
|  | Pygmies | Etho |  | 0.149 | 0.0425 | | 0.2194 | | 0.2444 | | 0.1018 | | 0.6256* | | 0.0829 | | 0.1123 | | 0.2447 | 0.2279 | 0.1538 |
| **Mixed Villages** | Pygmies | Doumassi |  |  | 0.0566 | | -0.065 | | 0.0205 | | -0.1233 | | 0.3 | | -0.116 | | -0.0695 | | -0.0362 | 0.1569 | -0.0353 |
|  | Pygmies | Esseng |  |  |  | | 0.0624 | | 0.0407 | | -0.008 | | 0.3573 | | 0.0069 | | 0.0127 | | 0.1368* | 0.0463 | 0.0943* |
|  | Pygmies | Mimbang |  |  |  | |  | | **-0.0511** | | -0.1204 | | 0.1904 | | -0.0753 | | -0.0744 | | -0.0187 | 0.1052 | -0.0012 |
|  | Bantus | Mimbang |  |  |  | |  | |  | | -0.056 | | 0.132 | | 0.0233 | | -0.0344 | | 0.0995 | 0.0023 | 0.1026 |
|  | Pygmies | Nkok Akom |  |  |  | |  | |  | |  | | **0.2553** | | -0.15 | | -0.1615 | | -0.0423 | 0.0962 | -0.0561 |
|  | Bantus | Nkok Akom |  |  |  | |  | |  | |  | |  | | 0.4194 | | 0.2258 | | 0.3832 | 0.2937 | 0.3746* |
|  | Pygmies | Zangaville |  |  |  | |  | |  | |  | |  | |  | | **-0.0792** | | -0.0905 | 0.1448 | -0.1003 |
|  | Bantus | Zangaville |  |  |  | |  | |  | |  | |  | |  | |  | | 0.0334 | 0.1059 | 0.0101 |
| **Forestry Villages** | Bantus | Bordamure |  |  |  | |  | |  | |  | |  | |  | |  | |  | 0.229* | -0.0209 |
|  | Bantus | STIBG |  |  |  | |  | |  | |  | |  | |  | |  | |  |  | 0.2086* |
